# Three-hinge gyri link individualized folding topology to cortical and superficial white-matter diffusion organization

**DOI:** 10.64898/2026.10.14.757725

**Published:** 2026-10-15

**Authors:** Minheng Chen, Xiuyu Dong, Jiale Cheng, Dan Hu, Tianming Liu, Dajiang Zhu, Gang Li

## Abstract

Individual differences in cortical folding are a hallmark of human brain anatomy, but what this variability reflects in the underlying tissue remains unclear. Three-hinge gyri (3HGs), where three gyral crests converge, provide an individualized higher-order folding landmark for probing this relationship. Using 7T diffusion MRI in six postmortem hemispheres, we found lower mean, axial, and radial diffusivity and higher fractional anisotropy at 3HGs than at matched gyral crowns, with the principal tensor pattern replicated in 414 age-matched *in vivo* adults. Diffusivity differences extended across the cortical ribbon, whereas anisotropy- and fiber-related differences strengthened toward and beneath the white surface. *Ex vivo* , diffusion measures also varied with gyral-skeleton degree, and streamlines sampled beneath 3HGs preferentially reached other 3HGs. These findings link individualized folding topology to cortical and superficial white-matter diffusion-inferred tissue properties, providing tissue-level context for understanding the microstructural correlates of individual folding variation.

